# Annexin A11 and TDP-43: core players in neurodegeneration

**DOI:** 10.1007/s00401-026-03043-0

**Published:** 2026-07-07

**Authors:** Courtney L. Smith, John L. Robinson, Edward B. Lee

**Affiliations:** Center for Neurodegenerative Disease Research, Department of Pathology and Laboratory Medicine, Institute On Aging, Perelman School of Medicine, University of Pennsylvania, 3rd Floor Maloney Building, 3400 Spruce Street, Philadelphia, PA 19104 USA

**Keywords:** Neurodegenerative disease, FTLD-TDP, ALS, Annexin A11, TDP-43

## Abstract

Annexin A11 (ANXA11) is a Ca^2^⁺-dependent phospholipid-binding protein that has recently emerged as a key player in neurodegeneration. Rare pathogenic *ANXA11* variants were initially identified in cases of amyotrophic lateral sclerosis (ALS). Since then, ANXA11 has been linked to a broader spectrum of related neurodegenerative diseases. Two independent studies demonstrated that ANXA11 co-aggregates with TDP-43 in all cases of frontotemporal lobar degeneration with TDP-43 pathology (FTLD-TDP) type C, with cryo-EM revealing heteromeric ANXA11–TDP-43 filaments. These discoveries support the direct pathological interaction between the two proteins as an important feature of FTLD-TDP type C. We also described secondary ANXA11 pathology in related neurodegenerative diseases, including limbic-predominant age-related TDP-43 encephalopathy (LATE), and more rarely in ALS and FTLD-TDP types A and B. ANXA11 and TDP-43 co-aggregates are also a feature of a FTLD-TDP associated with primary lateral sclerosis. These advances have renewed interest in ANXA11 as a major player in ALS/FTLD pathogenesis in both genetic and sporadic neurodegenerative diseases. In this review, we summarize ANXA11 pathology across genetic and sporadic cases, highlighting its heterogeneous overlap with TDP-43 pathology. We synthesize current knowledge of ANXA11’s physiological roles in phase separation, membrane repair, and RNA granule dynamics, integrating emerging evidence on how disruption of these processes may promote pathological aggregation and toxicity. Finally, we outline priorities for future research, with particular emphasis on elucidating ANXA11’s mechanistic connection to TDP-43.

## Introduction

Frontotemporal degeneration (FTD) is a progressive neurodegenerative disease characterized by atrophy of the frontal and temporal lobes, leading to diverse clinical spectrum including debilitating behavioral changes and language deficits [54, 71, 110]. Frontotemporal lobar degeneration with TDP-43 pathology (FTLD-TDP) is a major pathological subtype of FTD, characterized by the predominantly cytoplasmic mislocalization and aggregation of the RNA-binding protein TDP-43. FTLD-TDP is further classified into subtypes (A-E) based on the distribution and morphology of TDP-43 inclusions [44, 81]. Among these, FTLD-TDP type C is characterized by the accumulation of long dystrophic neurites in the superficial cortical layers, accompanied by sparse neuronal cytoplasmic inclusions [44, 53]. FTLD-TDP type C often manifests as the semantic variant of primary progressive aphasia and is predominantly sporadic, without known genetic linkage [81].

Annexin A11 (ANXA11) is a Ca^2^⁺-dependent phospholipid-binding protein with rare genetic variants linked to familial and sporadic cases of amyotrophic lateral sclerosis (ALS), with or without accompanying FTD [91, 101, 111]. Unexpectedly, two landmark studies discovered ANXA11 co-aggregation with TDP-43 in all cases of FTLD-TDP type C [3, 80]. Secondary ANXA11 pathology was also found, albeit more rarely, in related TDP-43 proteinopathies, including ALS and limbic-predominant age-related TDP-43 encephalopathy (LATE) [80]. Further, ANXA11-TDP-43 heteromeric filaments were identified in three cases of FTLD-TDP type C, indicating co-accumulation in disease [3]. These discoveries implicate ANXA11 as a key player in FTLD-TDP type C and potentially broader TDP-43-related neurodegenerative diseases, extending its relevance beyond the rare genetic cases. These results have now been extended to describe a novel pathological sub-group of FTLD-TDP associated with FTLD-PLS [28, 68, 99].

In this review, we introduce ANXA11, detailing its unique structural features and physiological roles in calcium signaling, RNA transport, and membrane trafficking, and we discuss the most-relevant experimental evidence implicating dysfunction in these pathways as being responsible for ANXA11-mediated dysfunction in ALS/FTD. We also explore future research directions, including ANXA11’s potential interaction with TDP-43 and its involvement in other neurodegenerative diseases. Beyond the scope of this review, ANXA11 is also linked to autoimmune diseases, including sarcoidosis, as well as increased metastasis in malignant cancers; these connections are covered in previous reviews [77, 106].

## ANXA11 structure

ANXA11 is a 505 amino acid protein encoded by the *ANXA11* gene with 16 exons, resulting in two major coding isoforms that exhibit broad expression across tissue types and development stages [6, 23]. The Annexin A (ANXA) protein family are Ca^2^⁺-regulated membrane-binding proteins (except ANXA10) with a conserved ANXA motif [26], also characterized by their association with RNA [75, 98]. ANXAs can be dissected into their highly conserved C-terminal and unique N-terminal domains [27].

**Fig. 1:**
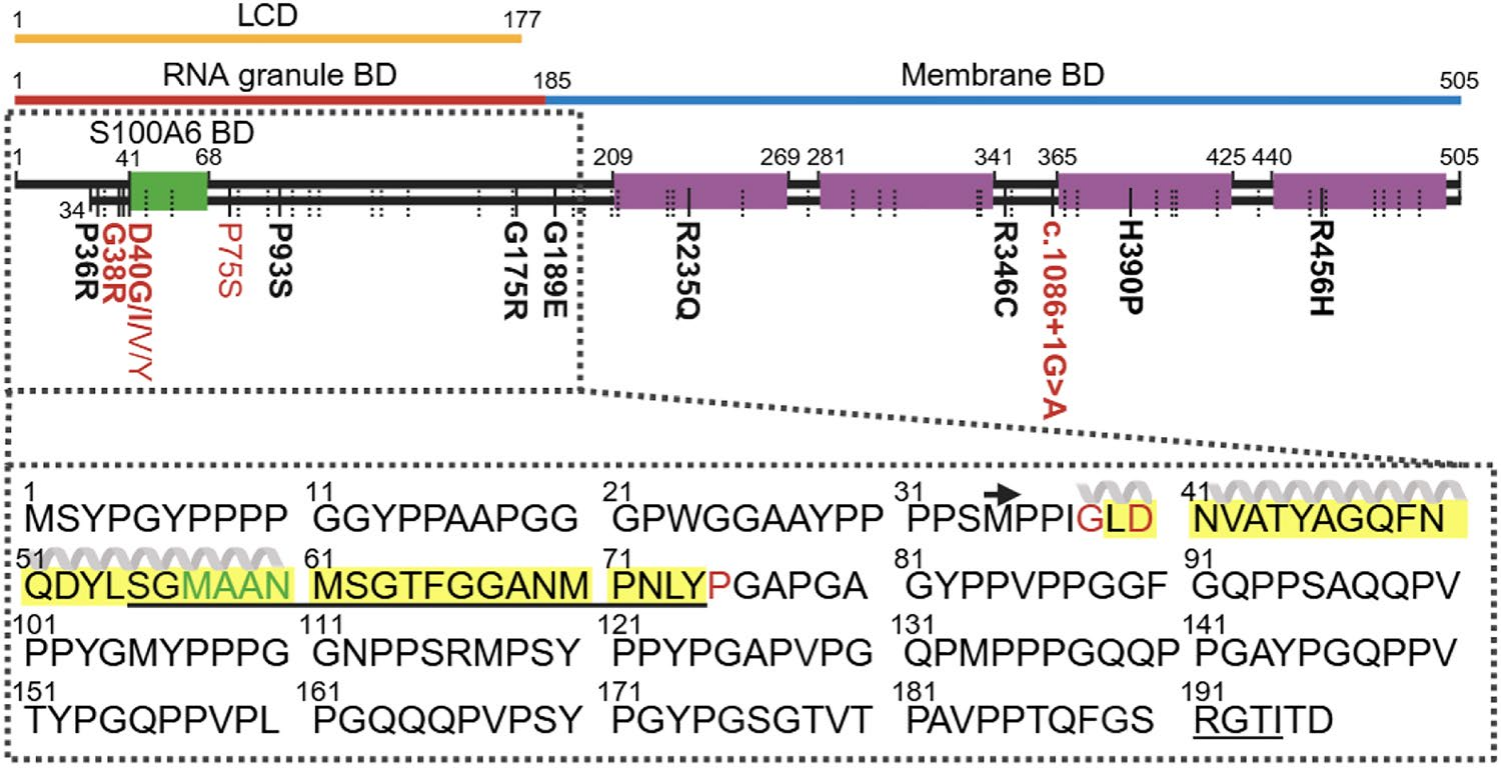
Schematic of Annexin A11 protein structure. ANXA11's interaction domains and known neurodegenerative disease-linked variants (top). *ANXA11* variants are indicated with vertical lines. Predicted amino acid changes are shown for variants with evidence of a disease-causing effect: variants with experimental evidence for their pathogenicity are bolded and those with pathological evidence are in red. The S100A6 binding domain is shown in green (N41-A68). The N-terminal LCD is enlarged to visualize the glycine- and proline-rich amino acid sequence (bottom). The alternative start site for the shorter ANXA11 peptide is denoted with an arrow (M34). The predicted alpha helical region is indicated with a gray helix (G38-N60) and positions disease-linked variants are shown in red. The residues identified in the heteromeric ANXA11-TDP-43 are highlighted in yellow (L39-Y74) with the amino acids at the interface with TDP-43 marked with a bold underline (S55-Y74). The RGTI motif that is predicted to stabilize the N-terminus is underlined (R191-I194). *LCD* low-complexity domain, *BD* binding domain

The ANXA C-terminal domain is comprised of four homologous annexin repeat domains (ARDs) (or eight in the case of Annexin A6), each approximately 70 amino acids which form five alpha-helices and a short beta sheet [10] (Fig. 1). The ARDs collectively form a disc-like secondary structure [48, 49], where Ca^2^⁺ ions interact with the loops between alpha helices I and VI on its convex side [27, 48]. Ca^2^⁺ binding does not cause a dramatic change in the ANXA11 secondary structure, rather it confers a positive charge to the convex surface of ANXA11, priming it for binding to negatively charged phospholipids [23, 47, 49]. Ca^2^⁺ binding also increases the thermal stability of mouse ANXA11 in vitro [41].

An in vitro study of rat ANXA11, 93.3% sequence identity of full-length protein and 96.5% sequence identity in C-terminal domain, identified a four amino acid motif (_191_RTGI_194_) preceding the first alpha-helix of the first ARD which interacts with several helices within the ANXA core domain, possibly stabilizing contact between domains I and VI [49]. Deletion of this motif significantly decreases the thermal stability of the C-terminal core. This RGTI motif is conserved across ANXAs, apart from ANXA1, ANXA8 and ANXA13 [49].

The C-terminal domain is highly conserved across the ANXA protein family; therefore, functional divergence is attributed to unique N-terminal domains [27]. The typically small, 10–30 amino acid N-terminal domain of annexins mediates protein–protein interactions and is the target of several posttranslational modifications. ANXA11 has the longest N-terminal domain in the ANXA family, which spans approximately 196 amino acids [27, 102]. This NTD is intrinsically disordered and prion-like, enriched in glycine (35/196), tyrosine (18/196), and proline residues (59/196), with a predicted helical segment spanning residues 38 to 59 [6, 23, 69, 91]. This unique composition enables ANXA11’s NTD to undergo liquid–liquid phase separation (LLPS) in vitro, forming droplets that facilitate its association with ribonucleoproteins (RNPs) and RNA granules [64]. A recent study demonstrated RNA-binding of several ANXAs, including ANXA11—this may also contribute to ANXA11s association with RNA granules; however, ANXA11 lacks an RNA-binding motif [75]. In vitro, full-length ANXA11 forms dynamic, reversible liquid-like droplets, with its N-terminal domain (amino acids 1–185) being both necessary and sufficient for this phase separation [47]. In contrast, the C-terminal domain (residues 186–505) does not contribute to LLPS and remains diffuse under similar conditions.

The N-terminal domain of ANXA11 also facilitates its binding to effector proteins, including ALG-2 (apoptosis-linked gene-2, encoded by *PDCD6*) [85] and S100A6 (Calcyclin) [62]. Yeast two-hybrid screens and in vitro assays confirmed the Ca^2^⁺-dependent interaction between ANXA11 and ALG-2, which is disrupted upon deletion of the N-terminal domain [85]. S100A6 dimers bind ANXA11 with high affinity between residues 52–68 and lower affinity between residues 41–48 [89] (Fig. 1). Hydrophobic residues L52, M55, A56, and M59 are critical for this interaction [95]. Alternative splicing of exon 1 of ANXA11 results in a truncated 472 amino acid protein which lacks first thirty-three amino acids and does not co-precipitate with S100A6 [6, 95].

Several observations point to the N-terminal domain of ANXA11 may be particularly relevant to neurodegeneration. First, this region shares structural and biophysical features with the low-complexity domains of TDP-43 and FUS, which influence their aggregation, phase separation, and toxicity [83]. Second, disease-associated variants appear to cluster within the N-terminal region, most notably at residues G38 and D40 [91, 101, 107]. Third, cryo-electron microscopy (cryo-EM) and biochemical analyses of FTLD-TDP type C tissue have shown that the filament core of ANXA11-TDP-43 heteromeric assemblies is comprised of N-terminal ANXA11 sequences, implicating this domain in pathological fibril formation [3, 80].

ANXA11 is widely expressed in the body, with high expression in excitatory neurons and microglia [92]. In dividing cells, ANXA11 localizes to the midbody and facilitates cytokinesis [103]. In non-dividing cells, such as those in the adult rat spinal cord, ANXA11 is predominantly cytoplasmic [55]. ANXA11 does not have a canonical nuclear localization signal, however, it has a predicted hydrophobic PY-NLS (residues 84–133) and thus may be a substrate for nuclear import by Karyopherin β2 [42]. Deletion studies identified a 60 amino acid segment within the N-terminal domain (residues 115-196) as necessary and sufficient for nuclear localization of rabbit ANXA11 [63]. The ANXA11 N-terminal fragment (residues 1–207) phenocopies the full-length protein with predominant nuclear localization, while a C-terminal fragment (residues 208–505) is lost from the nucleus [63]. The intracellular localization of ANXA11 is regulated by the Ca^2^⁺ concentration, demonstrating dynamic shifts in association with the nuclear envelope and plasma membrane under conditions of elevated Ca^2^⁺ ions [104].

In summary, ANXA11 is a Ca^2^⁺-binding protein whose intracellular localization is dependent on cellular Ca^2^⁺ levels and developmental stages. Its modular structure provides insights into its involvement in cellular processes, such as membrane trafficking, laying the foundation for understanding the effects of neurodegenerative disease-associated ANXA11 variants.

## ANXA11 in neurodegenerative diseases

### Genetic link to neurodegeneration

Genetic variants in *ANXA11* were linked to ALS in 2017 [91]. Subsequent studies identified variants in the *ANXA11* across diverse ALS and FTD cohorts [52, 65, 72, 84, 96, 101, 111]. Beyond ALS/FTD, *ANXA11* variants have been implicated in a range of neurodegenerative and neuromuscular diseases, including corticobasal syndrome [92], multisystem proteinopathies [46], as well as related neuromuscular diseases inclusion body myopathy [36] and progressive childhood-onset oculopharyngeal muscular dystrophy [66]. We reported the *ANXA11* p.P75S variant in a patient presenting with progressive supranuclear palsy with features of frontotemporal disease [80]. Another study identified *ANXA11* p.D40G in six cases of semantic variant primary progressive aphasia [45]. This phenotypic pleiotropy is reminiscent of other genes linked to the ALS/FTD disease spectrum, such as *VCP* [86].

With expanded genetic testing, detection of single-nucleotide polymorphisms (SNPs) in patient populations has risen. A central challenge in the field is to distinguish benign from pathogenic variants. According to gnomAD, a large public database that compiles human genetic variation data from many large-scale sequencing projects, *ANXA11* missense and frameshift variants are present in the unaffected population, suggesting many are well tolerated and not disease causing. Instead, rare *ANXA11* genetic variants may predispose carriers to ANXA11 aggregation. These genetic variants may offer clues into how ANXA11 dysfunction could drive to toxicity in disease. Disease-linked *ANXA11* variants show phenotypic heterogeneity and variable pathological overlap with TDP-43. This variability raises important questions about how these variants impact 1) ANXA11 function in neurons, and 2) its association/co-aggregation with TDP-43 in disease. Neurodegenerative disease-linked *ANXA11* variants are illustrated in Fig. 1, and pathological (Table 1) and experimental (Table 2) evidence and supporting their pathogenicity are synthesized below.

**Table 1:** Summary of Annexin A11 neuropathology in patient tissue

	Diagnosis (number of cases examined)	Tissue	Pathological description	Co-localization with TDP-43	Analysis type	References
*Genetic cases*						
p.G38R	ALS-FTD (1)	Midfrontal, motor cortex, hippocampus	NCIs and DNs	Rare	Double-IHC	[101]
		Superior temporal isocortices, caudate nucleus, mesencephalon	NCIs, DNs and torpedo-like neurites	Rare		
		Dentate nucleus	Skein-like NCIs	No		
		Spinal cord	Skein-like and tubular-shaped and round NCIs	Occasional		
p.G38R	ALS-FTD (1)	Spinal cord	Skein-like NCIs	Not assessed	IHC; IF	[56]
		Motor cortex	NCIs and DNs			
p.G38R	ALS (1)	Motor cortex, hippocampus, striatum	NCIs	Yes	IHC; IF; histology	[80]
		Spinal cord	Skein-like NCIs	Occasional		
p.D40G	ALS (1)	Spinal cord	Skein-like, filamentous, tubular-shaped, and basket-like NCIs	No	IHC; IF	[91]
		Motor cortex	DNs; torpedo-like neuritic structures			
		Temporal cortex	NCIs			
		Dentate gyrus	NCIs			
		Occipital cortex	NCIs and DNs			
p.D40I	Oculopharyngeal MD (1)	Muscle	Sarcoplasmic small aggregates	Not assessed	IHC; IF; histology	[66]
			Large vacuole-related aggregates			
			Sarcolemma aggregates			
p.D40Y	MSP (3)	Muscle	Macrophage cytoplasmic inclusions in necrotic muscle fibers	Occasional	IHC; IF; histology	[46]
			Rod- or torpedo-like NCIs			
			Intravacuolar inclusions			
p.D40Y	MD (3)	Muscle	Intravacuolar accumulations	Not assessed	IHC	[36]
p.D40V	MD (1)	Muscle	Necrotic muscle fibers	Yes	IHC; IF; histology	[57]
			Rimmed vacuoles with ANXA11 aggregates			
			Cytoplasmic TDP-43- and ANXA11-positive inclusions			
p.P75S	PSP-FTD (1)	Neocortex, striatum	DNs	Absent	IHC; IF; histology	[80]
		Hippocampus	NCIs and DNs			
		Pons	Globular NCIs			
c.1086 + 1G > A	ALS (1)	Spinal cord	Skein-like NCIs	Partial	IF	[84]
		Motor cortex	Round, small NCIs	Absent		
*Sporadic cohorts*						
	FTLD-TDP type C (34)	Whole brain	NCIs and large DNs	Yes	IHC; IF; immunoblot	[80]
	AD-LATE (8)	Amygdala; hippocampus	globular NCIs, DNs	Predominant	IHC; IF	
	ALS (2)	Whole brain	NCIs and DNs	Predominant		
	FTLD-TDP type A (2)	Whole brain	Compact NCIs, DNs	Predominant	IHC; IF; immunoblot	
	FTLD-TDP type B (1)	Whole brain	NCIs and DNs	Predominant	IHC; IF; immunoblot	
	FTLD-TDP type C (9)	Frontal and temporal cortex	NCIs and large DNs	Yes	IF, double immuno-EM	[3]
	FTLD-TDP type C (7)	Bilateral inferior frontal gyrus	NCIs and DNs	Yes	IHC, IF	[38]
	FTLD-TDP type C (4)	Motor cortex, midbrain, and spinal cord	NCIs and DNs	Not assessed	IHC	[109]
		Frontal and temporal cortex			Immunoblot, immuno-EM	
	FTLD-TDP type C (32)	Whole hemisphere, cortical and subcortical regions	NCIs and DNs	Yes	IHC, IF	[37]
	FTLD-TDP type C (23)	Frontal and temporal cortex, hippocampus, basal ganglia, and medulla	Long DNs	Yes	IHC; IF	[68]
	FTLD-TDP unclassifiable (FTLD-PLS) (TAP)	Frontal and temporal cortex, hippocampus, basal ganglia, and medulla	Compact crescent- or ring-like NCIs circling the nucleus, compact oval bodies, DNs	Predominant	IHC, IF	
	FTLD-TDP (C9orf72 repeat expansion carriers) (limited TAP)	Temporal cortex	Compact crescent- or ring-like NCIs circling the nucleus, tangle-like neurites, DNs	Limited	IHC, IF	
	FTLD-TDP type C (42)	Basal forebrain, motor cortex, medulla, spinal cord, middle frontal gyrus, posterior hippocampus, medulla	NCIs and DNs	Yes	IHC, IF	[28]
	FTLD-TDP (TAP) (13)	Same as above	NCIs and DNs, Compact crescent- or ring-like NCIs circling the nucleus, neurites characteristic of several subtypes	Predominantly	IHC, IF	
	FTLD-TDP (limited TAP) (19)	Same as above	Pleomorphic NCIs and limited DNs; granular NCIs in deep cortical regions	Predominantly	IHC, IF	
	FTLD-PLS (6)	Same as above	Long, rope-like DNs; infrequent Pick body-like NCIs	Predominantly	IHC, IF	
	FTLD-PLS (4)	Frontal cortex	NCIs and short DNs	Yes	IHC, IF, immunoblot, double immuno-EM	[99]

The number of cases examined in each study is included in parentheses

*ALS* amyotrophic lateral sclerosis, *FTD* frontotemporal degeneration, *MD* muscular dystrophy, *MSP* multisystem proteinopathy, *PSP* progressive supranuclear palsy, *FTLD* frontotemporal lobar degeneration, *AD* Alzheimer’s disease, *LATE* limbic-predominant age-related TDP-43 encephalopathy, *PLS* primary lateral sclerosis, *TAP* TDP-43 and ANXA11 proteinopathy, *IHC* immunohistochemistry, *IF* immunofluorescence, *EM* electron microcopy, *NCIs* neuronal cytoplasmic inclusions, *DNs* dystrophic neurites

**Table 2:** Summary of Annexin A11 variant functional analysis in cellular and animal models

Amino acid/nucleotide change	Functional defect	Model	Source
p.P36R	Motor deficits starting at 10 months in heterozygous and homozygous knock-in mice, muscle fiber abnormalities, motor neuron loss, ANXA11 and TDP-43 aggregation, impaired autophagic flux	Mouse	[51]
p.G38R	Aberrant axonal morphology, decreased axonal ANXA11 puncta, disrupted association with the nuclear envelope, defects in nuclear morphology	Zebrafish	[56]
	Impaired stress granule disassembly	HELA cells	[64]
p.D40G	Impaired phase transitioning after heat shock	U2OS cells	[47]
	Impaired axonal transport of RNA granules	Primary neurons, zebrafish	
	Aberrant axonal morphology, decreased axonal ANXA11 puncta, disrupted association with the nuclear envelope	Zebrafish	[56]
	Impaired stress granule disassembly	HELA cells	[64]
	Decreased immunoprecipitation with S100A6	HEK293T cells	[91]
p.D40I	Impaired stress granule disassembly	Patient fibroblasts	[66]
p.P93S	Decreased ANXA11 association with lysosomes, impaired axonal RNA transport	hiPSC-derived neurons	[92]
p.G175R	Incomplete rescue of motor defect in knockdown *drosophila*; motor defect and shortened lifespan when coexpressed with hTDP-43	*Drosophila*	[7]
p.G189E	Decreased immunoprecipitation with S100A6	HEK293T cells	[91]
p.R235Q	Aggregation in larval neurons; motor defect and shortened lifespan when coexpressed with hTDP-43	*Drosophila*	[7]
	Impaired phase transitioning after heat shock	U2OS cells	[47]
	Decreased association with lysosomes, impaired axonal transport of RNA granules	Primary neurons	
	Impaired axonal transport of RNA granules	Zebrafish	
	Aberrant cytoplasmic foci morphology	Mouse primary motor neurons	[91]
	Increased insolubility, decreased immunoprecipitation with S100A6	HEK293T cells	
	Increased insolubility, sequesters wild-type ANXA11 into cytoplasmic aggregates	SH-SY5Y cells	
p.R346C	Impaired phase transitioning after heat shock	U2OS cells	[47]
	Decreased association with lysosomes	Primary neurons	
	Impaired axonal transport of RNA granules	Primary neurons, zebrafish	
c.1086 + 1G > A	Cytoplasmic aggregation, increased insolubility	HEK293T cells	[84]
p.H390P	Decreased Ca^2+^-dependent association with nuclear membrane	Patient fibroblasts, HELA cells	[64]
	Impaired stress granule disassembly	HELA cells	
p.R456H	Decreased Ca^2+^-dependent association with nuclear membrane	Patient fibroblasts, HELA cells	[64]
	Impaired stress granule disassembly	HELA cells	
Knockdown (shRNA)	Impaired axonal transport of RNA granules	Primary neurons	[47]
Knockdown (siRNA)	Disruptions in the early secretory pathway (ER-Golgi transport)	HT1080 cells	[88]
Knockdown (siRNA)	Age-dependent climbing defect, shortened lifespan	*Drosophila*	[56]
Knockout (CRISPR/Cas9)	Aberrant axonal morphology, neuromuscular junction defects, increased neuronal apoptosis, semi penetrant motor defects	Zebrafish	

*hTDP-43* human TDP-43, *ER* endoplasmic reticulum, *Golgi* Golgi apparatus

### ANXA11 pathology in genetic cases of neurodegeneration

**Fig. 2:**
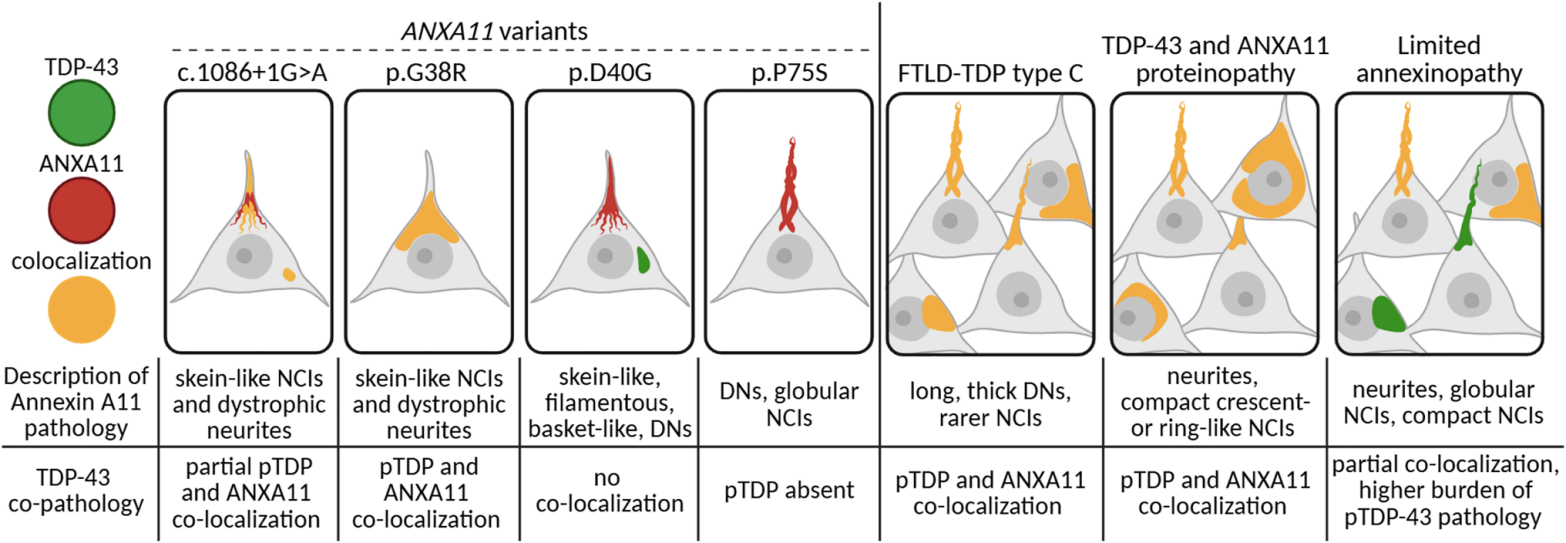
Characteristics of Annexin A11 and TDP-43 pathology. Pathology in cases with *ANXA11* variants (left) and non-variant cases (right). Independent TDP-43 pathology is represented with green, ANXA11 pathology is shown in red, and TDP-43-ANXA11 co-pathology is represented with yellow. ANXA11 pathology and the pattern of co-localization with TDP-43 are described in Table 1.

ANXA11 pathology has now been confirmed across several independent cohorts, using multiple techniques including immunohistochemistry, immunofluorescence, immunoblot, and immuno-EM from brain, spinal cord, and muscle extracts (Table 1). In most cases with *ANXA11* variants, ANXA11 pathology is accompanied by TDP-43 proteinopathy (Fig. 2), but the pathological relationship varies widely: in some cases,the pathologies appear completely independent; in others, they are present in the same individual but are non-overlapping; in some, they co-occur in select tissues; and in still others, they co-localize in all affected regions. Below, we review the distribution of ANXA11 pathology in published variant studies and report on the co-occurrence of TDP-43 pathology.

Pathology has been described in three individuals with the *ANXA11* p.G38R variant [56, 80, 101]. Teyssou et al. observed ANXA11 accumulations in the form of NCIs and neurites in midfrontal cortex, motor cortex, dentate gyrus, caudate, spinal cord, and dentate nucleus. In the dentate nucleus and spinal cord, the NCIs were larger and more filamentous. TDP-43 pathology was similarly abundant, with accumulations of both proteins in the midfrontal cortex, motor cortex, and dentate gyrus; however, co-localization was not noted. In spinal cord, ANXA11 NCIs were observed to be independent of TDP-43 inclusions in neurons with and without nuclear TDP-43 immunoreactivity, though some co-localization was also observed [101]. Marchica et al. reported abundant ANXA11 NCIs and neurites in motor cortex and occasional skein-like NCIs in spinal cord. They also observed diffuse cytoplasmic ANXA11 in control motor neurons, but TDP-43 pathology was not assessed [56]. We described an *ANXA11* p.G38R case with abundant compact NCIs in the motor cortex, hippocampus, and striatum with rare skein-like inclusions in the spinal cord [80]. These aggregates were ubiquitinated, and there was frequent co-localization with TDP-43 in most areas of the brain. However, in spinal cord tissue, some ANXA11 and TDP-43 inclusions overlapped, but TDP-43-positive ANXA11-negative aggregates were also observed.

*ANXA11* p.D40G variant pathology was described by Smith et al. ANXA11 pathology was primarily limited to motor neurons, though a mild burden of NCIs and neurites was noted in the dentate gyrus and temporal cortex. In motor neurons, NCIs were described as skein-like, tubular-shaped, filamentous, and occasionally basket-like. TDP-43 inclusions were found in the same regions, but the results of double fluorescent labelling were only reported in spinal cord, where aggregates were found to be non-overlapping yet often present in the same or neighboring motor neurons [91].

*ANXA11* D40I variant pathology was described by Natera-de Benito et al. ANXA11 immunostaining of muscle biopsy revealed ANXA11 aggregates in both the sarcoplasm and sarcolemma of fibers. Aggregates varied in size and location: ~ 60% were sarcoplasmic small aggregates, ~ 30% were sarcoplasmic large aggregates, and ~ 10% were located only in the sarcolemma. Confocal imaging revealed ANXA11 aggregates shaped like pearl strips with varied sizes in the sarcoplasm and as layered subsarcolemmal chains. TDP-43 pathology was not investigated in this study [66].

*ANXA11* D40Y variant pathology was described in two individuals [36, 46]. Johari et al. described ANXA11 accumulations in rimmed vacuolar fibers in muscle, but TDP-43 immunolabelling was not performed. Leoni et al. reported rod- or torpedo-like ANXA11 cytoplasmic inclusions. Additionally, ANXA11 positive inclusions were found in the cytoplasm of macrophages invading necrotic muscle fibers. Serial sections showed both ANXA11- and TDP-43-positive intravacuolar accumulations. ANXA11-positive inclusions were more prevalent, and ~ 50% were also positive for TDP-43, while ~ 75% of the TDP-43 inclusions were ANXA11-positive. A similar case harboring a D40V variant reported rimmed vacuoles with ANXA11 and TDP-43-positive aggregates in muscle tissue [57].

*ANXA11 *P75S variant pathology was described in a case of vacuolar annexinopathy by Robinson et al. There were abundant ANXA11 aggregates across multiple brain areas, including neocortex and hippocampus. The majority of ANXA11 aggregates were neuritic inclusions, with a more severe burden in the striatum where distinct neuronal vacuolization was present. NCIs with a jagged appearance were also described in the nuclei of the basis pontis. ANXA11 aggregates in areas with a more severe burden were ubiquitinated, but none were positive for TDP-43 [80]. It will be interesting to see if additional cases of vacuolar annexinopathy emerge in additional cohorts, and whether this pattern represents a distinct subclass of cases with primary ANXA11 pathology.

### ANXA11 pathology in non-variant cases of neurodegeneration

Remarkably, ANXA11 pathology occurs most frequently in non-variant cases. Multiple studies have reported ANXA11 and TDP-43 co-pathology in all FTLD-TDP type C cases [3, 28, 37, 38, 68, 80, 109]. Together, these studies characterize a pattern of ANXA11 pathology marked by the accumulation of ANXA11 inclusions with TDP-43, as well as the presence of sarkosyl-insoluble N-terminal ANXA11 fragments. To date, there is seemingly complete co-occurrence of the two pathologies, including in the large neurites characteristic of type C cases, but also in smaller neurites and NCIs. ANXA11- and TDP-43-positive dystrophic neurites also partially co-localize with the dendritic marker MAP2, suggesting these neurites are derived from neuronal dendrites [38]. ANXA11 and TDP-43 co-pathology is also present in upper motor neurons as well as anterior horn cells of the spinal cord in FTLD-TDP type C cases with motor involvement [109].

Another pathological subgroup has emerged with ANXA11 and TDP-43 co-pathology associated with FTLD with primary lateral sclerosis (PLS). Although the reported cohorts are small, this subtype appears to be predominantly male and presents with compact, pleomorphic NCIs that can appear crescent- or ring-like around the nucleus [28, 68, 99]. This new subgroup suggests ANXA11 and TDP-43 pathology may show a predilection for upper motor neurons.

ANXA11 inclusions are also present in cases of Alzheimer’s disease with LATE-NC, ALS, and FTLD-TDP types A and B, including cases with *C9orf72* expansions, and *GBA* and *TBK1* variants [80]. The overall incidence of ANXA11 pathology in these non-FTLD-TDP type C sporadic cases was approximately 5 to 10%. In these cases, ANXA11 pathology adopted the morphology of the primary TDP-43 pathology. The distribution of ANXA11 inclusions also matched that of TDP-43 inclusions, although the overlap was incomplete; ANXA11 pathology is best described as a subset of TDP-43 pathology. This limited ANXA11 pathology appears to show some association with *C9orf72 *expansions and* TBK1 *variants [28, 68].

#### ANXA11-TDP-43 heteromeric filaments

Building upon their prior resolution of monomeric TDP-43 filament structures in FTLD-TDP type A and ALS/FTLD-TDP type B, Arseni and colleagues utilized electron cryo-EM to describe the structure of insoluble filaments isolated from the prefrontal and temporal cortices of three FTLD-TDP type C cases [3]. Unlike the monomeric TDP-43 filaments observed in FTLD-TDP type A and type B, they unexpectedly found that FTLD-TDP type C filaments were heteromeric, made up of TDP-43 co-assembled with ANXA11 [1–3]. The TDP-43-ANXA11 filaments displayed a left-handed helical twist, distinct from the right-handed twist of filaments in types A and B, consisting of residues G228/G284–N345 of TDP-43 and ANXA11 residues S39–Y74. The filament fold includes an interface between the antiparallel chains of TDP-43 Q327-N345 and ANXA11 S55-Y74. The FTLD-TDP type A and type B folds are incompatible with ANXA11 binding. The presence of these heteromeric filaments, and the lack of monomeric filaments of either ANXA11 or TDP-43 alone, indicates that these proteins may co-assemble in FTLD-TDP type C. ANXA11 and TDP-43 fibrils have since been characterized by immunoelectron microscopy in four additional FTLD-TDP type C cases with motor involvement [109].

#### Insoluble ANXA11 fragments

Biochemical analyses also revealed a unique ~ 23 kDa ANXA11 fragment (full-length ANXA11 is ~ 56 kDa) in FTLD-TDP type C cases and cases with *ANXA11* mutations [3, 80]. The ~ 23 kDa ANXA11 fragments should correspond to approximately 190 amino acids. Arseni and colleagues proposed that the fragment must include residues L39–Y74 to facilitate filament formation. At the time, our group suggested the fragment must include at least 180–250 residues, in part based on an antibody epitope that were listed by the commercial vendors for one of the antibodies used to characterize this fragment. However, since our publication, we independently determined that the antibody we used to detect the C-terminal domain actually binds to an N-terminal ANXA11 epitope (data not shown). The company has since updated this antibody’s epitope to residues 1-200 (Abcam, ab236599). Given this information, we agree with Arseni and colleagues that the ~23 kDa fragment is comprised of ~190 amino acids in the N-terminal domain of ANXA11, although mass spectrometry analyses will be necessary to precisely define the fragment's composition. The shared identification of this ~ 23 kDa ANXA11 fragment points to its potential as a disease biomarker and a focal point for future research.

## ANXA11 pathomechanisms of toxicity

The presence of neurodegenerative disease-linked *ANXA11* variants, together with the discovery of ANXA11 inclusions in all cases of FTLD-TDP type C, implicates ANXA11 in neurodegeneration. Further, the aggregation of ANXA11 in rare cases of FTLD-U with *ANXA11* variants underscores its potential to cause disease independently of TDP-43. In this section, we explore potential mechanisms of ANXA11-mediated toxicity, which likely reflect a convergence of loss-of-function and gain-of-function pathways. We discuss relevant findings from pathology and genetic models and emphasize the relevance of each disease mechanism to TDP-43 pathobiology.

### ANXA11 loss-of-function

Given ANXA11's broad involvement in essential cellular pathways, ANXA11-mediated toxicity may arise from its decreased expression, stability, or function. Evidence from in silico and cellular models supports the idea that disease-associated variants destabilize ANXA11’s structure. N-terminal variants (G38R and D40G) are predicted to disrupt the amphipathic helix [91], although this is not supported by recent structural analysis [23]. In contrast, several C-terminal variants (R235Q, R302C, R346C, and G491R) are predicted to disrupt salt bridges within the core domain, compromising structural integrity [49]. Consistent with these predictions, patient-derived fibroblasts harboring the ANXA11-D40I variant and mesenchymal stem cells with the G228Lfs* variant exhibit reduced ANXA11 protein levels [64, 66].

ANXA11 loss appears to cause dysfunction in critical cellular processes. In primary neurons, ANXA11 knockdown impairs mRNA granule dynamics, decreasing their co-transport with lysosomes and reducing mRNA localization at growth cones [47]. ANXA11 knockdown results in age-dependent motor defects as well as defects in axonal growth and neuromuscular junction formation in *Drosophila* and zebrafish [7, 56]. These data suggest that disease-linked ANXA11 variants may confer loss-of-function toxicity via decreased stability or expression and loss of ANXA11 is detrimental to neuronal function.

### ANXA11 gain-of-function

Spatially, TDP-43 and ANXA11 pathology in FTLD-TDP type C cases coincides with areas of high ANXA11 expression in control tissue, including widespread neuronal staining and bilaminar cortical distribution [37] (Protein Atlas, https://www.proteinatlas.org). ANXA11 protein levels are elevated in the temporal pole region and in pyramidal neurons, where ANXA11 localizes to dendritic shafts and frequently co-localizes with MAP2 [37, 38]. This correlation raises the possibility that elevated ANXA11 levels contribute to regional vulnerability. This may contribute to the vulnerability of upper motor neurons in FTLD-PLS with ANXA11 pathology, as pyramidal neurons in L2 of the sensorimotor cortex have high expression of ANXA11 [37]. Consistent with this hypothesis, proteomic analysis of FTLD-TDP type C cases revealed increased ANXA11 levels in the temporal cortex, but not the dentate gyrus [60], whereas no corresponding change in ANXA11 at the transcript level [78]. Together, these findings suggest that differences in post-transcriptional mechanisms, such as impaired protein turnover, may contribute to the selective enrichment of ANXA11 in disease-relevant regions.

ANXA11 toxicity may also arise from its aggregation and fibrillization. Recombinant ANXA11 displays an intrinsic propensity to aggregation, like other proteins low-complexity domains (LCDs), such as TDP-43 and FUS [64]. In vitro, the N-terminal LCD of ANXA11 (residues 2–196) forms monomeric ribbon-like fibrils that are resistant to proteinase K digestion and positive for amyloid markers [89]. Shorter fragments containing residues 2–88 and 2–68 retain fibril-forming ability, whereas residues 2–52 are insufficient to nucleate fibrils. Proteinase K mapping further identifies residues 27–40, 47–57, 69–90, and 171–191 as potential components of ANXA11 filament cores [89]. These observations indicate that the N-terminal LCD contains multiple aggregation-prone segments capable of driving filament assembly.

Disease-associated variants can modulate aggregation properties of ANXA11. Variants G38R and D40G within the N-terminal LCD significantly enhance precipitation of full-length ANXA11 in vitro, whereas C-terminal variants such as H390P and R456H have little measurable effect on aggregation under similar conditions [64]. N-terminal fragments containing G38R, D40G, P175S, or A189T form fibrils comparable to wild-type ANXA11, but exhibit slower fibrillization kinetics, possibly due to impaired nucleation [89]. The C-terminal variant R235Q accumulates in the insoluble fraction when overexpressed in cells [91]. Together, these findings suggest that aggregation is an intrinsic property of ANXA11 and can be modulated by disease-linked variants.

Several disease-linked variants in the N-terminal LCD introduce charged residues into this aggregation-prone region. For example, G38R and D40G exchange a small, uncharged amino acid with a larger, charged amino acid—this may alter local electrostatic interactions and fibril assembly. Proline residues can regulate aggregation of proteins with low-complexity domains, as their rigid backbone structure disrupts β-sheet formation [105]. Interestingly, three disease-associated variants in the N-terminal LCD affect proline residues: P71L, P75S, and P93S. Substitution of proline with leucine or serine residue may increase local conformation flexibility and extend the region of ANXA11 compatible with forming beta sheets.

The pathogenic relevance of ANXA11 aggregation is further supported by evidence that aggregates can sequester functional ANXA11. The *ANXA11* R235Q variant forms insoluble cytoplasmic aggregates in primary motor neurons that recruit wild-type ANXA11 [91]. These data suggest ANXA11 aggregation may perpetuate further aggregation and ANXA11 loss-of-function. This sequestration model resembles models proposed for TDP-43, in which aggregates exacerbate toxicity by reducing the availability of functional protein [43]. Loss of function due to reduced protein stability and sequestration may collectively amplify ANXA11’s contribution to neurodegenerative pathology. Counter to this, however, is that the levels of full-length, soluble ANXA11 appeared to be unchanged in cases with annexinopathy, including FTLD-TDP type C [80].

### Post-translational modificationsand cleavage

Post-translational modifications (PTMs) are regulators of protein function, localization, and interaction. Non-physiological and abnormal PTMs are a common feature of aggregating proteins in neurodegenerative disease, including Aβ, tau, and TDP-43 [15, 94]. Similarly, PTMs of ANXA11 may contribute to its functional dysregulation and aggregation in ALS/FTD, however, disease-relevant ANXA11 PTMs remain undefined. Early studies demonstrated general ANXA11 phosphorylation, but did not resolve the modified residues [25, 61]. Phosphorylated ANXA11 exhibits reduced vesicle membrane binding, suggesting phosphorylation may regulate ANXA11’s physiological function [61]. Phosphorylation of other ANXAs can alter their affinity for Ca^2^⁺ ions, increase their susceptibility to proteolytic cleavage, and trigger their secretion [27]. Whether similar modifications in ANXA11 influence its phase separation or susceptibility to aggregation warrants further investigation.

Cleavage of ANXA11 may represent another mechanism of dysregulation in disease. The discovery of a 23 kDa ANXA11 fragment in FTLD-TDP type C cases raises questions about ANXA11-targeting proteases, its cleavage site(s), as well as the relevance to neurodegeneration [3, 80]. It is unclear whether this cleavage is a byproduct of abnormal accumulation or occurs at an earlier time point and has an active role in disease. ANXA11 does not possess a KFERQ-like motif for chaperone-mediated autophagy but contains a strong PEST region at its N-terminus [8]. PEST regions are rich in proline (P), glutamate (E), serine (S), and threonine (T) and are associated with protease targeting and rapid turnover [79].

Future research should focus on identifying disease-specific ANXA11 PTMs and cleavage patterns, determining their physiological and pathological roles. For instance, whether ANXA11 cleavage or phosphorylation alters its phase separation or propensity to aggregate. From a therapeutic perspective, targeting these processes—by inhibiting pathological cleavage events or modulating PTMs—may restore ANXA11 function and prevent aggregation-related toxicity. Disease-specific PTMs or cleavage products could also serve as biomarkers for ANXA11-related neurodegeneration, perhaps to identify cases FTLD-TDP type C.

### Calcium dynamics

Calcium ions (Ca^2^⁺) serve as essential intracellular messengers, regulating diverse processes including membrane trafficking, autophagy, and cellular repair [5, 11]. In neurons, precise regulation of intracellular Ca^2^⁺ levels is critical for synaptic plasticity, neural network function, and overall cellular homeostasis [14]. Calcium release is also triggered by membrane damage, including lysosomal damage (discussed further below). Dysregulation of Ca^2^⁺ homeostasis is a well-established feature in ALS/FTD, with mechanisms such as glutamate hyperexcitability contributing to chronic Ca^2^⁺ imbalance [14].

ANXA11 and its binding partners play crucial roles in responding to intracellular Ca^2^⁺ signaling, and there is some evidence that ANXA11 dysfunction can significantly affect Ca^2^⁺ homeostasis. Studies in fibroblasts and mesenchymal stem cells derived from patients with ALS-linked *ANXA11* variants (e.g., G38R, H390P, R456H, and G228Lfs*29) show increased basal cytoplasmic Ca^2^⁺ levels and reduced ER Ca^2^⁺ release, suggesting impaired intracellular Ca^2^⁺ storage and signaling [64]. Molecular dynamics simulations predict that ALS-linked ANXA11 variants in the ARDs, H390P and R456H, have decreased affinity for Ca^2^⁺ [64]. Disrupted ANXA11- Ca^2^⁺ dynamics may affect downstream membrane trafficking and resealing. Further, Ca^2^⁺-free ANXA11 may be more aggregation prone, as suggested by in vitro analysis [23].

### S100A6 binding

S100A6 is a small EF-hand Ca^2^⁺-binding protein within the S100 family, central to Ca^2^⁺ signal transduction. Upon Ca^2^⁺ binding, S100A6 undergoes a conformational change, exposing a hydrophobic surface that facilitates interactions with various target proteins [21]. These interactions regulate diverse cellular processes, including stress responses, vesicular transport and secretion, apoptosis, and protein homeostasis. S100A6 is expressed in neurons and astrocytes, and its dysregulation has been linked to several neurodegenerative diseases. S100A6 levels are elevated in reactive astrocytes in Alzheimer’s disease, as well as ALS lumbar and thoracic spinal cords [34, 35]. This astrocytic expression may be a non-specific stress-related response as S100A6 is also elevated in stressed wild-type mice [9]. However, secreted S100A6 has been shown to negatively affect neurons by reducing neurite complexity and protein turnover, potentially exacerbating neurodegeneration [9].

The functional significance of ANXA11-S100A6 interaction in ALS/FTD is poorly understood. ANXA11 variants have been shown to affect S100A6 binding, although this was not replicated in another study [89, 91]. This is significant because S100A6 expression prevents aggregation of the ALS-linked ANXA11-R235Q variant in HEK293 cells [91]. This effect is reversed with proteasome inhibition, suggesting S100A6 may promote the targeting of mutant ANXA11 to the ubiquitin–proteasome pathway [91]. Notably, S100A6 also interacts with CACYBP/SIP as part of the ubiquitin ligase pathway [59]. In vitro, S100A6 can dissolve pre-formed ANXA11 monomeric fibrils, suggesting a proteasome-independent mechanism [89]. Whether S100A6 affects the heteromeric ANXA11-TDP-43 fibrils found in ALS/FTD pathology remains to be tested.

The interaction represents an intriguing avenue for therapeutic development as a potential modifier of ANXA11 phase-separation and aggregation behavior. However, S100A6’s dual roles—protective in some contexts and potentially harmful in others—highlight the need for a nuanced understanding of its functions across different cellular environments and mutation backgrounds.

### Membrane binding

ANXA11 interacts with intracellular membranes via its C-terminal annexin repeat domain. Ca^2^⁺ binding to this domain increases its positive surface charge, enhancing its affinity for negatively charged phospholipids, which are abundant in lysosome and ER membranes and play critical roles in recruiting trafficking complexes like COPII [47, 58]. Once bound to membranes, ANXA11 can induce phase changes that reduce phospholipid motility and increase membrane order [69]. This effect can be modulated by ANXA11’s interacting proteins: ALG-2 enhances ANXA11-mediated membrane condensation, increasing membrane stiffness, while S100A6 has the opposite effect [69]. ANXA11 and its binding partners participate in several membrane trafficking and repair pathways that are relevant to neurodegeneration.

#### ER-Golgi trafficking

Efficient trafficking of membrane and secretory proteins relies on the proper functioning of the endoplasmic reticulum (ER) to Golgi apparatus (Golgi) transport pathway. ALG-2 links Ca^2^⁺ signaling to ER-Golgi trafficking through its interaction with ANXA11. In the presence of Ca^2^⁺, ALG-2 recruits ANXA11 to ER exit sites (ERES), where ANXA11 stabilizes the association of SEC31A with these sites, a critical step in COPII vesicle formation [40]. Reduced ALG-2 or ANXA11-ALG-2 interaction leads to the redistribution of ERES and increased trafficking through the secretory pathway, signaling network destabilization [88]. These disruptions are associated with ER stress, Golgi fragmentation and altered receptor trafficking—key hallmarks of ALS/FTD and related neurodegenerative diseases [16, 31, 88]. While ALS-linked *ANXA11* variants have not been shown to affect ER-Golgi trafficking or ALG-2 binding [91], it is tempting to speculate that ANXA11 aggregation or decreased ANXA11 activity may interfere with these processes. ANXA11 aggregation may also sequester its binding partners, including SEC31A, reducing normal trafficking to the plasma membrane and contributing to cellular stress.

#### Autophagy

Beyond FTLD-TDP type C, more limited ANXA11-TDP-43 co-pathology appears to be enriched in cases with *C9orf72* expansions and *TBK1 *variants [28, 68]. *C9orf72* expansions are most commonly found in FTLD-TDP type B cases with or without ALS, where toxicity is thought to arise from a combination of C9orf72 loss-of-function, as well as the production of toxic repeat RNA and dipeptide repeat proteins (DPRs) [50]. TBK1 (TANK-binding kinase 1) is a serine/threonine kinase that plays important roles in inflammation and selective autophagy, where it phosphorylates several autophagy adaptors, including OPTN and p62 [70].

These two genetic causes of ALS/FTD converge on the endolysosomal and autophagic pathways. In C9orf72-associated disease, DPR proteins can sequester TBK1 in patient tissue, impairing its kinase activity and disrupting autophagosome maturation and cargo clearance [87]. An ALS/FTD-linked TBK1 variant has been shown to cause autophagolysosomal dysfunction in neurons [13]. The increased prevalence of ANXA11 pathology in *C9orf72* expansion and *TBK1* variant cases raises the possibility that ANXA11 may also intersect with this pathway. Supporting this idea, ANXA11-p.P36R-expressing mice exhibit signs of autophagy impairment, including accumulation of autophagic markers and abnormal vesicular structures [51]. In this way, ANXA11 aggregation or loss-of-function could contribute to impaired autophagy. Alternatively, the enrichment of ANXA11 pathology in these cases may reflect more severe underlying proteotoxic stress, in which primary disruptions in autophagy or TDP-43 homeostasis may secondarily promote ANXA11 aggregation.

#### Membrane repair

ANXA11, together with the other members of the annexin family, plays a critical role in Ca^2^⁺-dependent membrane repair [12]. Upon membrane injury and Ca^2^⁺ efflux through the damaged membrane, ANXA11 and related annexins (e.g., ANXA7) rapidly localize to the site of damage, forming a repair scaffold that stabilizes the membrane and prevents further loss of cellular integrity [32]. The low-complexity domains of annexins promote condensate formation at sites of membrane damage, which likely serves as a rapid protective response, analogous to the behavior of stress granules [17, 93]. ANXA11 subsequently recruits its binding partner ALG-2, initiating ESCRT-III-mediated membrane repair, including the recruitment of CHMP2B—another ALS/FTD-linked protein [32]. Deletion of the ANXA11 low-complexity domain, as well as introduction of select C-terminal variants (R235Q and R456H), disrupts recruitment to sites of membrane injury, while other variants (G38R, P93S, R346C) do not alter this localization [32].

ANXA11’s role in membrane repair is particularly relevant in the context of oxidative stress-linked membrane damage, as well as nuclear and lysosomal injury. Oxidative stress is strongly implicated in ALS pathogenesis and can promote lipid peroxidation, leading to altered membrane integrity and biophysical properties [33, 74, 97, 100]. Because ANXA11 membrane recruitment depends on interactions with phospholipids, lipid peroxidation may influence its localization or repair functions. However, the effects of oxidative membrane damage on ANXA11 biology have not been directly examined. Further studies will be needed to determine whether oxidative stress alters ANXA11 membrane recruitment or contributes to ANXA11 pathology.

Nuclear injury—arising from nucleoporin sequestration [19], impaired nuclear envelope surveillance and repair [20, 22], or cytoskeleton-driven damage [18, 29, 73]is well documented in ALS/FTD models and patient tissue [24]. Additionally, aberrant nuclear morphology has been observed in zebrafish models of ANXA11 dysfunction as well as in the spinal cord of an ALS patient carrying the ANXA11-G38R variant [56]. ANXA11 localizes to the nuclear envelope in response to Ca^2^⁺ influx [104]. It is possible that ANXA11 may contribute to nuclear envelope repair, potentially through recruitment of the ESCRT-III complex.

Lysosomal dysfunction is increasingly recognized as a hallmark of ALS/FTD pathology. Lysosomal injury can arise from multiple disease-associated processes, including lipid dysregulation and physical disruption by aggregate formation [82]. Given ANXA11’s association with lysosomal membranes, it is plausible that it may participate in lysosomal membrane surveillance or repair [47]. ANXA11 has been reported to co-localize with stress granule components on lysosomal membranes—this interaction has been proposed to facilitate stress granule transport [47]. However, these observations were made under conditions of substantial cellular stress. An alternative interpretation is that lysosomal membranes were damaged by the stressor and that ANXA11, together with stress granule proteins, was recruited as part of a rapid membrane repair response to stabilize the damaged membrane. In this context, ANXA11 dysfunction may weaken both nuclear and lysosomal membrane repair pathways, resulting in persistent membrane instability and toxicity in ALS/FTD.

### Physiological and pathological phase transitions

Liquid–liquid phase separation (LLPS) is the physical demixing of molecules from a homogenous phase into dynamic, liquid-like droplets. This process enables cellular compartmentalization and localized biochemical reactions outside of membrane-bound organelles [90]. However, under certain conditions, phase-separated droplets can mature into more gel-like or solid states, potentially leading to irreversible aggregation [4, 108]. Such transitions have been implicated in the pathological aggregation of several RNA-binding proteins in neurodegenerative diseases, including TDP-43 and FUS in ALS/FTD [4, 76]. Similar mechanisms have been proposed for ANXA11.

Recombinant ANXA11 undergoes LLPS in vitro, forming liquid-like droplets stabilized by ionic and hydrophobic interactions within its N-terminal LCD [39, 47]. These droplets are dynamic under basal conditions but can become more stable over time. Several ALS/FTD-associated variants appear to alter this behavior. For example, the D40G variant forms fewer, but more stable LLPS droplets that have an increased tendency to aggregate, whereas the D40I variant further reduces droplet formation and promotes fibrillization [66]. In cell-based models, the R235Q variant enhances LLPS and slows droplet disassembly [47]. It remains an open question whether ANXA11 cleavage or post-translational modifications modulate its phase-separation behavior. Dysregulation of ANXA11 LLPS may impair the dynamics of RNA granules or promote the transition from condensate toward more stable aggregates.

#### Stress granules

Stress granules are transient membraneless organelles that form during cellular stress to sequester non-essential RNA, enabling stress adaptation and recovery. ANXA11 has been reported to localize to stress granules, where it can interact with RNA-binding proteins including FUS, G3BP1 and TIA1 [64]. Disrupted stress granule dynamics have been widely proposed as a disease mechanism in ALS/FTD, although their connection to TDP-43 aggregation remains unclear [67].

Several ANXA11 variants have been reported to affect stress granule dynamics in cell models. For example, the R235Q variant was shown to prolong stress granule retention in U2OS cells, resulting in delayed stress granule disassembly following stress removal [47]. Similarly, fibroblasts derived from patients carrying the D40G or D40I variant exhibited delayed stress granule disassembly [66, 96]. These findings suggest that altered ANXA11 LLPS may influence stress granule persistence. Because these experiments rely on acute stress induction to promote stress granule formation, the physiological relevance of these interactions in unstressed or pathological contexts remains unclear.

#### RNA granule transport

ANXA11 has been described as a molecular tether that links RNA granules to lysosomes, facilitating their co-transport along axons. In this model, ANXA11’s NTD interacts with RNA granules through phase separation, while its C-terminal domain binds lysosomal membrane [47]. Consistent with this model, several ANXA11 variants have been reported to impair RNA granule trafficking. In stressed primary neurons and zebrafish models, the D40G variant reduces stress granule motility and decreases β-actin mRNA localization at growth cones [47]. Similarly, the P93S variant exhibits reduced lysosome association in induced pluripotent stem cell-derived neurons, leading to decreased axonal transport of β-actin RNA [92]. Additional variants, including R235Q and R346C, reduce the number of RNA granules trafficking on lysosomes in axons and decrease β-actin mRNA at the growth cone. In vivo imaging in zebrafish neurons further demonstrated reduced RNA motility in live neurons [47]. These findings suggest that ANXA11 variants can disrupt RNA trafficking.

However, many of these experiments were performed under conditions of acute cellular stress, which strongly promotes stress granule formation and may alter lysosomal dynamics. Under these conditions, the observed co-localization of ANXA11, lysosomes, and stress granule components could reflect processes other than constitutive RNA granule transport. As discussed above, ANXA11 may be recruited to lysosomes as part of a maintenance or repair response, with stress granules co-localizing at sites of lysosomal damage. Further experiments under physiological conditions will be important to determine whether RNA granule-lysosome tethering represents a central function of ANXA11.

#### ANXA11 and TDP-43 co-aggregation

The discovery of heteromeric amyloid fibrils provides a direct structural link between ANXA11 and TDP-43 in FTLD-TDP type C [3]. However, ANXA11 and TDP-43 heteromeric filaments have yet to be replicated by cryo-EM in additional cohorts. It remains unclear whether these fibrils are pathogenic or protective, and whether they are specific to FLTD-TDP type C or perhaps are a part of normal aging. With this caveat, the discovery of heteromeric filaments also raises the possibility that ANXA11 or TDP-43 initiate the aggregation of the other protein, in a seeding or templating mechanism.

Neuropathological observations indicate that ANXA11 aggregation can occur independently of TDP-43 in certain genetic contexts, including with the p.P75S variant [80]. This observation leaves open the possibility that ANXA11 aggregation may precede or facilitate TDP-43 pathology in some contexts. Interestingly, the P75S variant occurs directly neighboring the region in ANXA11’s N-terminal domain that interfaces with TDP-43 in heteromeric filament core. Such variants may alter ANXA11 structural compatibility with TDP-43, potentially favoring ANXA11-dominant aggregation. It will be important to see whether disrupting the interface of ANXA11 and TDP-43 could mitigate heteromeric filament formation. Further biochemical and structural studies are needed to clarify whether ANXA11 alone can form amyloid-like fibrils and how these assemblies may interact with TDP-43. Cryo-EM analyses of patient-derived material, particularly from genetic cases, may help define the structural determinants of ANXA11-dependent filament formation.

### Mechanism summary

The presence of neurodegenerative disease-linked *ANXA11* variants, coupled with the discovery of ANXA11 pathology in FTLD-TDP type C and FTLD-PLS, strongly implicates ANXA11 in neurodegeneration. Given that *ANXA11* variants and pathology are linked to multiple neurodegenerative diseases, their contribution to disease is likely complex and context dependent. For example, in FTLD-TDP type C, ANXA11 may play an active role in neurodegeneration with TDP-43, whereas in related conditions such as ALS and LATE-NC, it might play a more secondary or modifying role. The variable contribution of ANXA11 to neurodegeneration may depend on expression, stability, and functional alterations.

## Discussion

The discovery of ANXA11-TDP-43 coaggregation in FLTD-TDP type C raises several important questions. First, what is the contribution of ANXA11 to disease? Is ANXA11 merely a bystander corrupted by TDP-43, or does it play a synergistic role that exacerbates disease progression? Second, what are the molecular mechanisms underpinning ANXA11-TDP-43 interactions? And under what cellular conditions—such as protease activity or specific PTMs—does ANXA11-TDP-43 fibril formation occur?

ANXA11 neuropathology is notable for the range of pathological contexts in which it is observed. In cases of FTLD-TDP type C and FTLD-PLS, ANXA11 and TDP-43 display nearly complete overlap [3, 28, 68, 80, 99]. In approximately 5% of LATE-NC, and more rarely in ALS and non-type C FTLD-TDP cases, ANXA11 co-localizes with a subset of TDP-43 inclusions [80]. In the ANXA11-P75S variant case, ANXA11 represents a primary proteinopathy that is independent of phosphorylated TDP-43 pathology. In other ANXA11 variants cases, ANXA11 pathology appears as an additional proteinopathy that intermingles with, but rarely co-localizes with, TDP-43 inclusions [80]. These patterns suggest that ANXA11 and TDP-43 pathologies exist on a spectrum: from coaggregation within shared filaments, to accumulation in the same neurons but in distinct aggregates, to entirely independent pathology. It will be important to determine whether additional rare FTLD-U cases exhibit ANXA11 pathology and whether such cases harbor ANXA11 variants. In addition, cryo-EM studies characterizing ANXA11 filaments across different pathological contexts, including in genetic variant cases, will provide valuable insights into structural heterogeneity within FTLD-TDP subtypes, ALS, and related diseases.

Varying mechanisms may account for the observed spectrum of ANXA11 and TDP-43 interactions. When the accumulation of ANXA11 and TDP-43 pathology occurs seemingly independent of each other, as in most genetic variant cases, ANXA11 loss-of-function may affect processes such as membrane trafficking or RNA granule transport, which might drive TDP-43 pathology. In cases of isolated ANXA11 pathology, such as in the P75S variant case, the serine substitution at proline 75 may stabilize ANXA11 self-assembly, preventing TDP-43 coaggregation. In LATE-NC and other sporadic ANXA11 cases, frequent co-localization of ANXA11 and TDP-43 may stem from shared upstream functions in stress granule assembly, RNA granule transport, or autophagy.

Unraveling these mechanisms will require robust experimental tools. Developing disease-specific ANXA11 antibodies will enable broader identification of ANXA11 pathology and isolation of fibrils for structural studies. A priority is identifying the ~23 kDa ANXA11 cleavage fragment in FTLD-TDP type C tissue. Further, in vitro* and *in vivo models that recapitulate heteromeric ANXA11-TDP-43 fibril formation will be instrumental. *Drosophila* models of genetic ANXA11 variants suggest that humanized TDP-43 may be necessary for ANXA11 toxicity [7]. In mice, ANXA11 p.P36R knock-in led to ANXA11 inclusions and cytoplasmic TDP-43 mislocalization starting at two months of age and late-onset motor dysfunction [51]. Notably, mouse models of other ANXAs display stress-dependent phenotypes; it will be interesting to see if ANXA11 models exhibit stress vulnerabilities [30].

Clinically, ANXA11 holds promise as both a biomarker and a therapeutic target. Therapies targeting ANXA11 might also prove effective at targeting ANXA11-TDP-43 heteromeric fibrils. However, given ANXA11’s essential roles in calcium signaling, membrane repair, and RNA granule transport, strategies must avoid disrupting its normal functions. Targeting disease-specific isoforms, PTMs, or fragments—possibly via interactors like S100A6—may offer a safer approach.

In summary, ANXA11 and TDP-43 are similar proteins: both contain unstructured low-complexity domains that facilitate their association with RNA, both associate with stress granules, and may be involved in axonal RNA transport. Rare mutations in their corresponding genes are linked to ALS/FTLD, and both proteins are found in insoluble, ubiquitinated aggregates in FTLD-TDP type C. From these similarities, an interesting storyline emerges where ANXA11 and TDP-43 may be co-conspirators who corrupt each other in neurodegenerative disease.

## Data Availability

No datasets were generated or analysed during the current study.
